# Application of Optical Genome Mapping for the Diagnosis and Risk Stratification of Myeloid and Lymphoid Malignancies

**DOI:** 10.3390/ijms26125763

**Published:** 2025-06-16

**Authors:** Lucía Ballesta-Alcaraz, Mónica Bernal, Jose Ramón Vilchez, Jorge Antonio Palacios, Pilar Jiménez, Pilar Garrido, Juan Francisco Gutiérrez-Bautista, Francisco Ruiz-Cabello

**Affiliations:** 1 Servicio de Análisis Clínicos e Inmunología, University Hospital Virgen de las Nieves, 18014 Granada, Spain; lucia.ballesta.sspa@juntadeandalucia.es (L.B.-A.); monica.bernal.sspa@juntadeandalucia.es (M.B.); joser.vilchez.sspa@juntadeandalucia.es (J.R.V.); mpilar.jimenez.sspa@juntadeandalucia.es (P.J.); fruizc@ugr.es (F.R.-C.); 2 Programa de Doctorado en Biomedicina, University of Granada, 18016 Granada, Spain; 3 Instituto de Investigación Biosanitaria de Granada (ibs.GRANADA), 18012 Granada, Spain; jorge_palacios_rodriguez@yahoo.es; 4 Servicio de Hematología, University Hospital Virgen de las Nieves, 18014 Granada, Spain; mariap.garrido.sspa@juntadeandalucia.es; 5 Departamento de Bioquímica, Biología Molecular e Inmunología III, University of Granada, 18016 Granada, Spain

**Keywords:** optical genome mapping, OGM, hematologic malignancies, chromosomal alterations, cryptic rearrangements

## Abstract

Optical genome mapping (OGM) is a novel, high-resolution technology for genome-wide detection of structural variants, offering clear advantages over conventional cytogenetics in hematologic malignancies. We applied OGM to a large cohort of patients with acute myeloid leukemia (AML), myelodysplastic syndromes (MDSs), and B-cell acute lymphoblastic leukemia (B-ALL) to evaluate its clinical utility. In AML and MDS, it revealed high-risk alterations such as deletions in 5q31–5q32 and 7q22, and cryptic fusions like *NUP98::NSD1* that were missed by karyotyping or FISH. It also identified chromoanagenesis, a catastrophic chromosomal event linked to poor prognosis and often undetectable by standard methods. In B-ALL, OGM uncovered clinically relevant deletions in *CDKN2A/B*, *PAX5*, and *IKZF1*, as well as high-risk ploidy changes like hypodiploidy and hyperdiploidy, all important for risk assessment and frequently underdetected. OGM not only refines diagnosis and improves risk stratification but can also uncover cryptic and complex genomic abnormalities. Our findings support its integration into routine diagnostics to enhance classification, guide treatment decisions, and improve patient outcomes.

## 1. Introduction

Genetic characterization plays a fundamental role in the clinical management of hematological malignancies. Advances in the understanding of molecular and cytogenetic alterations underlying oncohematologic processes have led to substantial revisions in disease classification. The most recent frameworks proposed by the World Health Organization (WHO) and the International Consensus Classification (ICC) underscore the critical importance of genetic abnormalities in defining the various entities of acute leukemias, both myeloid and lymphoid [1,2,3,4]. In parallel, the European LeukemiaNet (ELN) recommendations establish genetic risk categories in acute myeloid leukemia (AML) based on specific cytogenetic and molecular aberrations. At the same time, the Molecular International Prognostic Scoring System (IPSS-M) integrates cytogenetic alterations and gene mutations to refine risk stratification in patients with myelodysplastic syndromes (MDS) [5,6]. These updated classifications incorporate revised genetic risk stratification models, updated response criteria, and new therapeutic guidelines [6]. As a result, the identification of genetic variants holds profound implications for phenotyping, prognosis, and treatment strategies in oncohematological diseases.

Currently, conventional karyotyping and fluorescence in situ hybridization (FISH) are considered the gold standard cytogenetic methodologies for detecting and identifying structural and numerical chromosomal variants, including duplications, deletions, inversions, translocations, insertions, and copy number variations (CNVs).

In this context, optical genome mapping (OGM) has emerged as a high-resolution method capable of detecting a wide range of structural variants in a single assay, including balanced and complex rearrangements that may be missed by other methods [7]. OGM enables the detection of structural anomalies—whether balanced or unbalanced, intrachromosomal or interchromosomal—with a theoretical resolution ranging from 500 base pairs to 5 kilobases, depending on the microchip used for genome assembly. Furthermore, recent technological advancements have extended its capabilities to include the identification of additional genomic phenomena such as uniparental disomy and loss of heterozygosity [8]. Therefore, OGM has been proposed as a high-resolution alternative that addresses the limitations of conventional cytogenetic techniques, enabling the comprehensive detection of chromosomal aberrations and structural variants in a single assay. This advancement may herald the advent of “next-generation cytogenetics” [9]. Moreover, OGM can be effectively complemented by NGS, which offers nucleotide-level resolution and facilitates the detection of single nucleotide variants (SNVs) and small insertions or deletions (indels). While OGM excels at identifying large-scale structural variants, NGS provides critical insights into disease-related genes, aiding in the interpretation of genomic alterations. The integration of these two technologies enhances diagnostic yield and enables more precise risk stratification by combining comprehensive structural analysis with detailed mutational profiling. This synergistic approach is particularly valuable in oncohematology, where both genomic architecture and sequence-level alterations play a pivotal role in guiding clinical decision-making [10].

Compared with other molecular techniques, such as DNA microarrays and multiplex ligation-dependent probe amplification (MLPA), OGM offers a more comprehensive and detailed genomic assessment. While DNA microarrays effectively detect copy number variations (CNVs) and loss of heterozygosity (LOH) [11], they cannot identify balanced structural rearrangements such as translocations or inversions. MLPA enables sensitive detection of specific deletions or duplications, particularly in leukemias and myelodysplastic syndromes [12], but lacks genome-wide resolution. In contrast, OGM detects a broad spectrum of structural variants, including balanced rearrangements and complex chromosomal architectures, in a single assay. This enhances diagnostic efficiency and reduces the need for multiple tests, positioning OGM as a superior alternative to array-based or probe-limited methods in hematologic malignancies [13].

This study presents a comprehensive analysis of the application of OGM in the diagnosis and characterization of a broad spectrum of hematological malignancies. The primary objective is to evaluate the clinical utility of OGM in comparison with conventional cytogenetic techniques, highlighting its potential to improve diagnostic accuracy, refine risk stratification, and identify novel therapeutic targets. Specifically, this work compares the performance of standard cytogenetic methods—such as FISH and conventional karyotyping—with that of OGM. The analysis encompasses various hematological malignancies and focuses on the ability of each method to detect structural variants. Importantly, some of these variants may offer critical insights for reassessing disease prognosis, tailoring treatment strategies, and ultimately enhancing clinical management for affected patients.

## 2. Results

### 2.1. Comparative Analysis of OGM, Karyotyping, and FISH in the Detection of Cytogenetic Abnormalities

For diagnostic and prognostic purposes, conventional karyotyping was performed in 97 out of the 114 patients included in this study. Among these, 64% showed results concordant with OGM. In 5% of cases, no cell growth was observed. In 21% of cases, although chromosomal alterations were detected by karyotyping, OGM more accurately characterized the affected chromosomal regions and/or identified a greater number of rearrangements, though these were not clinically relevant. Additionally, in 10% of cases, karyotyping failed to detect recurrent or clinically significant alterations related to the patients’ pathologies, which OGM identified.

The FISH technique was performed on 110 patients out of the 114 included initially in the study. In 67% of cases, FISH and OGM results were concordant. In 27.5% of cases, OGM identified chromosomal alterations that FISH could not detect due to the lack of specific probes for these abnormalities. Furthermore, in 5.5% of cases, FISH failed to detect alterations despite the presence of specific probes, including two *MYH11::CBFB* fusions, two del(5q), one del(7q), and one del(17p), all of which were successfully identified by OGM.

OGM analysis identified cytogenetic alterations in all samples analyzed. Of these, 69 samples (60%) exhibited recurrent or clinically relevant alterations associated with the disease under study, while 50 samples (43.8%) showed structural variants of uncertain clinical significance (VUS).

Several of the clinically significant alterations identified by OGM are further explored in detail in subsequent sections of this work.

### 2.2. OGM-Identified Alterations with Prognostic and Risk Classification Relevance

The use of OGM enabled the detection of several cytogenetic alterations with established prognostic significance and relevance for risk stratification. Many of these abnormalities, which were often cryptic or undetectable by conventional techniques such as karyotyping or FISH, provided valuable insights for patient management.

- Detection of Clinically Relevant Chromosomal Alterations in Myeloid Neoplasms by OGM

In a patient diagnosed with AML with myelodysplasia-related changes (AML-MRC) carrying adverse-risk mutations in *U2AF1* and *BCOR* (Patient 1, Table 1), OGM detected a partial duplication of the KMT2A gene at 11q23, with a variant allele frequency (VAF) of 86%, consistent with KMT2A-PTD. This alteration was not identified by FISH or conventional karyotyping but was confirmed through CNV analysis using NGS (Supplementary Table S1).

**Figure 1:**
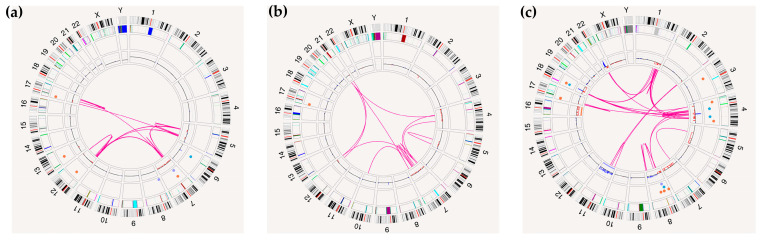
Optical genome mapping results illustrating cytogenetic complexity profiles: (**a**) Circos plot of a patient with acute myeloid leukemia (AML) associated with myelodysplasia. Deletions in 17p and 7q are indicated, along with multiple rearrangements involving chromosomes 5, 7, 12, and 17, consistent with a complex karyotype. Findings from optical genome mapping (OGM) together with a *TP53* mutation confirmed by next-generation sequencing (NGS) indicate biallelic inactivation of *TP53*; (**b**) Circos plot of a patient with myelodysplastic syndrome (MDS). The analysis revealed a ~1 Mb deletion in 17p encompassing the *TP53* gene, detected by OGM but not identified by conventional karyotyping or FISH. This deletion, in combination with a *TP53* mutation detected by NGS, supports biallelic inactivation of the gene. Additional abnormalities include monosomy seven and deletion of 5q; (**c**) Circos plot of a patient with therapy-related acute myeloid leukemia (t-AML). A deletion in 17p, a large deletion affecting nearly the entire chromosome 7, a deletion in 5q, and complex rearrangements involving chromosomes 1, 4, 8, and 18 were observed. As in the previous cases, the presence of a *TP53* mutation identified by NGS, together with the 17p deletion, indicates biallelic inactivation of *TP53*. From the outer to the inner rings of the circos plot, the following layers are displayed: (1) chromosomes and cytobands, (2) masked regions (heterochromatic, difficult to analyze), (3) structural variant (SV) region, (4) copy number variation (CNV) profile, and (5) in the center, inter- and intrachromosomal translocations represented as pink lines connecting the involved genomic loci. SVs < 1 Mb were filtered out from the circos plot unless they involved clinically significant regions. All alterations depicted in the circos plots are summarized in Supplementary Table S2.

OGM also identified a deletion at 7q22 in another patient (Patient 2, Table 1), with a VAF of 28%, which was undetectable by conventional karyotyping or FISH. This approximately 2 Mb region corresponds to one of the most commonly deleted regions (CDRs) on 7q observed in MDS and AML (Figure 1). NGS CNV analysis confirmed the finding, demonstrating the loss of one copy of the *CUX1* gene located at 7q22 (Supplementary Table S1).

In a separate AML patient (Patient 3, Table 1), FISH analysis using a break-apart probe specific for the *CBFB* gene (16q22) revealed a signal pattern indicative of a deletion in 70% of the interphase nuclei, which was also confirmed by karyotyping. However, OGM detected both the loss of *CBFB* (VAF = 68%) and a rearrangement between 16p13.11 and 16q22.1, resulting in the *MYH11::CBFB* fusion (VAF = 44%). This fusion, recognized as a favorable risk factor in AML, was not detected by FISH nor by karyotyping but was confirmed by quantitative PCR, which quantified the transcript at 100%.

**Figure 2:**
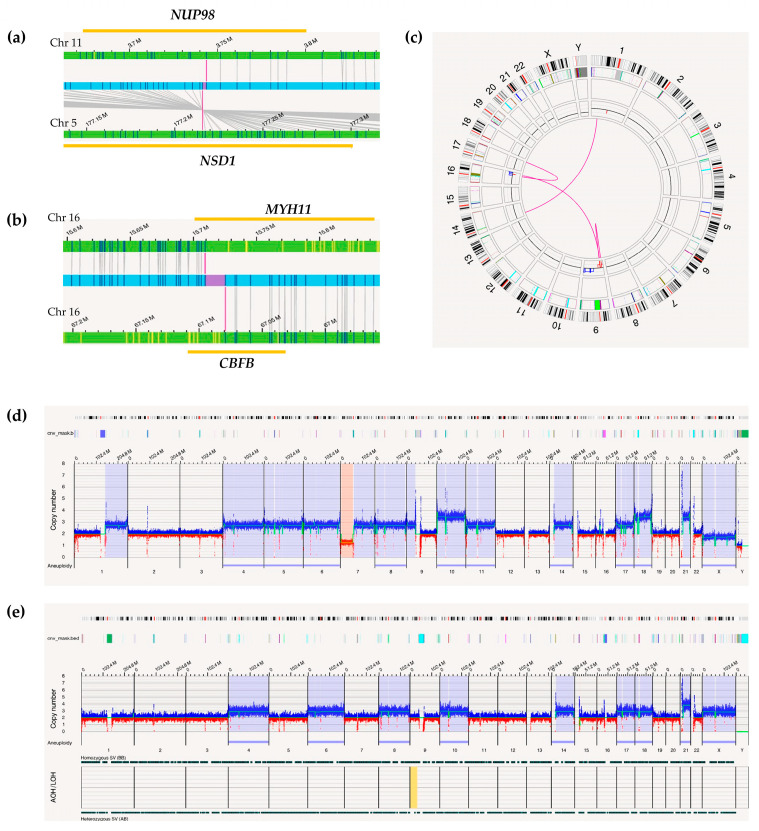
Visualization of structural alterations by optical mapping in hematological neoplasms: (**a**) Genome browser image of patient nº 4 with acute myeloid leukemia (AML). Optical genome mapping (OGM) revealed the translocation t(5;11)(q35;p15.5), resulting in the *NUP98::NSD1* gene fusion; (**b**) Genome browser image of patient nº 3 with AML. OGM detected a rearrangement between regions 16p13.11 and 16q22.1, leading to the *MYH11::CBFB* fusion; (**c**) Circos plot of patient nº 6 with T-cell acute lymphoblastic leukemia (T-ALL). OGM revealed several structural variants not detected by conventional karyotyping or FISH, including a deletion in 9p involving loss of *CDKN2A/B* and a t(1;14) translocation resulting in a *TAL1::TRD* fusion; (**d**) Whole genome view of patient nº 8 with B-cell acute lymphoblastic leukemia (B-ALL). A 55 Mb deletion in the short arm of chromosome 7 affecting the *IKZF1* gene was identified, along with multiple chromosomal gains, suggesting a hyperdiploid profile consistent with a complex karyotype; (**e**) Whole genome view using De Novo Assembly algorithm, patient nº 10 with B-ALL and Li-Fraumeni syndrome. High hyperdiploidy was detected, including trisomies of chromosomes X, 4, 6, 8, 10, 14, 17, and 18, as well as tetrasomy of chromosome 21. Secondary analysis showed no loss of heterozygosity, confirming true hyperdiploidy and ruling out masked hypodiploidy.

In three AML cases, including one pediatric patient (Patient 4, Table 1) and two adult patients, conventional cytogenetic techniques did not reveal any chromosomal alterations. However, OGM identified a reciprocal translocation between chromosomes 5 and 11, resulting in a fusion of the *NUP98* gene on 11p15 with the NSD1 gene on 5q35 (Figure 2). This cryptic alteration, associated with poor prognosis, was confirmed by NGS using a targeted fusion panel (Supplementary Table S1). Furthermore, NGS identified mutations in *WT1* and *FLT3*-ITD, commonly associated with this rearrangement.

Overall, the use of OGM facilitated the detection of clinically relevant genomic alterations in cases where conventional cytogenetic methods, such as karyotyping and FISH, were limited. These included cryptic structural variants, such as *KMT2A*-PTD and *NUP98::NSD1* fusions, microdeletions in commonly deleted regions of 7q, and more complex rearrangements, like 16q22 deletion (associated with the *CBFB* gene) and *MYH11::CBFB* fusion. All of these alterations have significant prognostic implications in myeloid neoplasms.

- OGM Accurately Characterizes Complex Genomic Rearrangements Associated with Chromoanagenesis in Myeloid Neoplasms

OGM identified complex genetic profiles in six patients with myeloid neoplasms, including two with AMLs with myelodysplasia-related changes (AML-MRC), one with post-cytotoxic therapy-related AML (AML-pCT), and three with MDS (one with increased blasts [MDS-IB], and two with low blasts [MDS-LB]. All showed complex rearrangements, including intrachromosomal and/or interchromosomal translocations, along with CNVs affecting a variable number of chromosomes, consistent with chromoanagenesis (Figure 1).

All patients, except for one with MDS-LB, exhibited alterations in *TP53*. Specifically, two patients with AML-MRC and one with MDS-LB showed a 17p deletion and a mutation in the remaining *TP53* allele, resulting in biallelic inactivation (Figure 1). The AML-pCT case showed a homozygous *TP53* mutation (VAF = 81.7%), indicating a copy-neutral loss of heterozygosity (CN-LOH) due to uniparental disomy. One patient with MDS-IB had a *TP53* mutation with a VAF of 57.9%, which was probably compatible with a CN-LOH in a small cellular subclone. On the other hand, the MDS-LB patient without *TP53* alterations displayed a less complex genetic profile, with rearrangements primarily affecting chromosome 5 (Figure 1). Notably, all patients exhibited deletions affecting chromosome 5 (5q) [one MDS-LB and one AML-Pct (Figure 1)], or chromosome 7 (−7/7q) (one AML-MRC), or both chromosomes (5q and −7/7q) (one MDS-LB, one MDS-IB, and one AML-MRC).

OGM results were fully concordant with FISH in four of the six patients. In MDS-LB, FISH did not identify chromosomal abnormalities; however, karyotyping revealed a deletion in 2q and a translocation between chromosomes 5 and 10. However, OGM detected several rearrangements affecting chromosome 5, including intrachromosomal fusions and interchromosomal translocations with chromosomes 2 and 10. As a result, interstitial deletions were observed in the long arm of chromosome 5, partially involving the commonly deleted region (CDR) (5q31–q32) on 5q in MDS. In another case (Patient 5, Table 1), karyotyping showed no abnormalities, but FISH detected a 5q deletion and monosomy 7, consistent with OGM results. OGM revealed chromoanagenesis, with complex rearrangements affecting chromosomes 4, 7, 12, and 21, primarily. Additionally, a microdeletion was identified in the 17p13.1 region, resulting in *TP53* loss (VAF = 19%), which FISH did not detect.

In summary, OGM enabled the precise identification of complex cytogenetic profiles associated with chromoanagenesis in patients with myeloid neoplasms. These profiles were primarily linked to progression or poor prognostic outcomes and were associated with *TP53* alterations. This information is crucial for genetic characterization and risk stratification in these patients.

- Uncovering Key Cytogenetic Markers and Risk Factors in ALL

One of the patients in the study was a pediatric case diagnosed with T-cell acute lymphoblastic leukemia (T-ALL) (Patient 6, Table 1). Conventional karyotyping and FISH did not detect any chromosomal abnormalities. However, OGM revealed multiple structural variants, resulting in a complex genomic profile. Notably, a 9p deletion led to the loss of *CDKN2A/B* genes (VAF = 29%) and a translocation between chromosomes 1 and 14 caused a *TAL1::TRD* gene fusion (VAF = 58%).

Six pediatric patients diagnosed with B-cell acute lymphoblastic leukemia (B-ALL) were also analyzed. In two cases, karyotyping did not identify any chromosomal abnormalities, with one showing a normal karyotype and the other lacking an informative result due to insufficient cellular growth (Patient 7, Table 1). FISH results were negative for all tested probes in both cases. However, OGM revealed an *IKZF1* gene deletion in each patient. In one case, a 50.7 Kb deletion exclusively affected this gene as an isolated anomaly. In the second patient, a 55 Mb deletion on 7p was observed, affecting *IKZF1*, and accompanied by a highly hyperdiploid profile with 60 chromosomes (Patient 7, Table 1) (Figure 2). Both the *IKZF1* deletion and hyperdiploidy were confirmed through CNV analysis using NGS (Supplementary Table S1).

Two additional B-ALL cases showed negative FISH results for all tested probes, although karyotyping revealed a deletion on the short arm of chromosome 9, among other alterations (Patient 8, Table 1). OGM confirmed these findings, precisely identifying a 9p deletion involving the *PAX5* and *CDKN2A/B* genes (Figure 2), alterations commonly observed in B-ALL.

In a fifth pediatric B-ALL patient (Patient 9, Table 1), the karyotype was reported as normal, while FISH using the *ETV6::RUNX1* fusion probe identified five copies of the *RUNX1* gene region per interphase nucleus. OGM detected an amplification (five copies) spanning a 22.2 Mb region on the long arm of chromosome 21 involving *RUNX1*, consistent with intrachromosomal amplification of chromosome 21 (iAMP21). This alteration was accompanied with deletions affecting the *CDKN2A/B* (9p21), *ETV6* (12p13), and *ERG* (21q22) genes in the patient’s sample.

Finally, a pediatric B-ALL case with a known diagnosis of Li-Fraumeni syndrome was analyzed (Patient 10, Table 1). Karyotyping revealed a profile of high hyperdiploidy, while FISH testing was negative for all probes, though gains of certain regions were observed. OGM characterized high hyperdiploidy with trisomies of chromosomes X, 4, 6, 8, 10, 14, 17, and 18, along with two additional copies of chromosome 21. In patients with Li-Fraumeni syndrome who develop hyperdiploid B-ALL, thorough cytogenetic evaluation is essential to exclude masked hypodiploidy. Accordingly, reanalysis using the “De Novo Assembly” OGM pipeline did not detect LOH in the altered regions, confirming true hyperdiploidy and ruling out the presence of a masked hypodiploid clone.

In conclusion, OGM is a valuable diagnostic tool for use in pediatric acute leukemias, offering more comprehensive analysis than traditional methods. Its ability to detect both CNVS and SVs in a single process helps to define the genomic complexity present in these patients, improving genetic diagnosis and risk stratification to enable better treatment decisions.

## 3. Discussion

In this study, we highlight the role of OGM as a key tool for the diagnosis and risk stratification in a large cohort of patients with various oncohematological malignancies, including AML, MDS, and B-ALL. Our findings demonstrate that OGM enables more precise diagnostic classification within the current WHO and ICC guidelines [1,2]. This improved classification can inform therapeutic decisions, such as eligibility for targeted therapies, inclusion in clinical trials, and hematopoietic stem cell transplantation. These results are consistent with previously published cohorts, where OGM revealed clinically relevant structural variants in 13–15% of cases that were missed by conventional cytogenetic techniques [15,16]. Our study supports these findings and advocates for incorporating OGM into diagnostic workflows.

A notable contribution of OGM in characterizing myeloid neoplasms is its ability to detect cryptic chromosomal abnormalities, such as KMT2A-PTD, a cryptic intragenic alteration [17] recognized as a high-risk molecular marker in MDS according to the IPSS-M scoring system [5]. This alteration can emerge during the progression from MDS to AML and is associated with treatment resistance and poor overall survival in secondary AML [18]. In our study, we identified this abnormality in a patient with AML with myelodysplasia-related changes (AML-MRC), where the presence of *KMT2A*-PTD, together with high-risk mutations, may have contributed to the leukemic transformation from an underlying MDS. OGM also identified a microdeletion affecting cytogenetic band 7q22 in an MDS patient, a region recognized as the most commonly deleted (CDR) among interstitial deletions of chromosome 7q [19]. This cytoband may represent a critical region, as it harbors several myeloid tumor suppressor genes, including *CUX1*, whose haploinsufficiency has been implicated in the pathogenesis of MDS and leukemogenesis [20,21]. However, further studies are needed to determine whether this alteration represents the minimal defining lesion for 7q deletions and to understand better its impact on the clinical course of patients affected by these deletions. These findings highlight the benefit of OGM’s superior resolution for detecting clinically significant small-scale variants.

OGM also detected the *NUP98::NSD1* fusion gene, resulting from the t(5;11)(q35;p15.5) chromosomal translocation [22]. This fusion is often undetectable by conventional cytogenetic methods due to its telomeric location [23,24]. The presence of *NUP98*::*NSD1* is associated with an adverse prognosis in AML, particularly when combined with the *FLT3* internal tandem duplication (*FLT3*-ITD) mutation [25,26]. In our cohort, OGM successfully identified this fusion in three AML patients, enabling accurate molecular characterization and reassessment of prognosis and treatment. This underscores OGM’s utility in detecting cryptic translocations.

In 5–10% of AML cases, inv(16)(p13q22) or t(16;16)(p13;q22) results in the *MYH11*::*CBFB* fusion, which is considered a favorable prognostic marker in the disease [27,28]. However, a less common event involving a 3’*CBFB* deletion (16q22.1) has been reported, which can occur at the inversion breakpoints [29,30,31,32]. AML with a 3’*CBFB* deletion/*CBFB* rearrangement often presents similar pathological features to AML with inv(16) but carries a higher risk of relapse, requiring hematopoietic stem cell transplantation [33]. In one AML patient from our study, the karyotype and FISH analysis identified only a 16q22 deletion, whereas OGM also detected the *MYH11*::*CBFB* fusion. The co-occurrence of inv(16)/t(16;16)(p13;q22) and *CBFB* deletion reflects complex genomic events that often remain undetected by conventional methods but are identifiable by OGM, thereby enhancing diagnostic precision and informing therapy.

Furthermore, OGM allowed the characterization of more complex cytogenetic profiles related to chromoanagenesis in six patients with AML or MDS. Chromoanagenesis is characterized by multiple catastrophic events, typically involving complex chromosomal rearrangements and copy number alterations affecting one or several chromosomes [34]. This phenomenon has a direct impact on the prognosis and progression of hematological neoplasms, being associated with poorer survival, more aggressive clinical behavior, and therapeutic resistance [35,36,37]. In this context, OGM emerges as a fundamental tool for further investigating chromoanagenesis in this group of disorders [38,39]. We observed that five patients showed biallelic inactivation of *TP53*, suggesting that multihit *TP53* alterations could be a key event associated with the chromoanagenesis process, consistent with previous studies [38]. OGM thus facilitates the detailed detection of complex structural patterns and provides insights into their prognostic relevance.

On the other hand, OGM also offers advantages compared with conventional methods for the cytogenetic evaluation of ALL [40]. T-ALL is characterized by genetic complexity that is not always well defined, as up to 30% of karyotypes fail to show cellular growth. In approximately another 30%, normal karyotypes are observed, even though chromosomal alterations may be detected using other techniques [41]. In our study, a pediatric patient with T-ALL presented normal karyotype and FISH results; however, OGM identified a complex genetic profile, including the *TAL1::TRD* gene fusion and deletions of the *CDKN2A/B* genes, alterations previously described in the literature [42,43].

B-ALL is also genetically complex, involving translocations, cryptic rearrangements, microdeletions, and aneuploidies [44,45], which typically require the combination of multiple methods such as karyotyping, FISH, MLPA (multiplex ligation-dependent probe amplification) or SNP (single-nucleotide polymorphism) array [46] OGM streamlines this process by detecting all relevant alterations in a single workflow to accurately characterize the genomic profile and enable proper classification of the specific B-ALL entity [1]. In our study, OGM accurately identified 9p deletions affecting *CDKN2A*, *CDKN2B*, and *PAX5* in B-ALL [47,48]. We also identified two pediatric patients with deletions at 7p12, resulting in *IKZF1* loss—a key event for risk reclassification and therapeutic decisions, as *IKZF1* deletions are associated with to worse outcomes [49]. In this context, OGM aids in detecting complex profiles, such as *IKZF1* Plus (co-occurring deletions in *CDKN2A/B*, *PAX5*, or *PAR1*), which are associated with a poor prognosis [50,51].

B-ALL with intrachromosomal amplification of chromosome 21 (iAMP21-ALL) is a high-risk entity (2% of all B-ALL) resulting from chromothripsis and typically involving *RUNX1* amplification and telomeric deletions [52]. Distinguishing iAMP21-ALL from favorable polysomy 21 can be difficult using FISH. In our cohort, OGM confirmed the iAMP21 diagnosis previously suggested by FISH, demonstrating its advantage in detecting high-risk features in B-ALL.

OGM also assessed the possibility of endoreduplicated hypodiploidy in a patient with Li-Fraumeni syndrome who developed B-ALL. Initially, OGM revealed a pattern of high hyperdiploidy, consistent with the karyotype and FISH results. In 60–65% of B-ALL cases with TP53 mutations, hypodiploid genomes can undergo endoreduplication, resulting in the presence of both hypodiploid and hyperdiploid (doubled) clones. This condition, known as ‘masked hypodiploidy’, is associated with an adverse prognosis [53]. OGM differentiated true hyperdiploidy from masked hypodiploidy using LOH analysis via the De Novo Assembly pipeline, classifying the disease risk as favorable.

Although OGM can identify numerous relevant alterations in hematological malignancies, its limitations must be acknowledged. Its sensitivity for detecting CNVs in minor clones is 15–20%, and it has difficulty in repetitive regions (centromeres, telomeres) where reference mapping is incomplete [54]. As with any genomic platform, sample quality, data interpretation, and integration with other diagnostics, such as NGS, are critical. Nonetheless, ongoing advancements are expected to reduce these limitations and further improve clinical utility.

Compared with techniques like DNA microarrays and MLPA, OGM offers a more comprehensive and unbiased view of the genome. While microarrays and MLPA are useful for targeted or copy number changes, they cannot detect balanced or complex structural variants. OGM, by contrast, enables genome-wide detection of a broad range of structural alterations—including balanced rearrangements and large insertions or deletions—within a single assay, streamlining diagnosis and reducing the need for multiple tests.

Finally, although this limitation did not directly affect our study population, a significant concern regarding the use of the human reference genome GRCh38 (hg38) is its disproportionate representation of individuals of European ancestry. This introduces population bias that can impact the accuracy of genomic analyses in non-European populations. The current reference does not adequately capture global genetic diversity, potentially leading to errors in variant identification and reduced effectiveness of genetic studies in underrepresented groups. To overcome these limitations, new approaches such as pangenomes are being developed, incorporating multiple genomes from diverse populations to provide a more comprehensive representation of human genetic variation [55].

In summary, our findings support the clinical utility of OGM in the diagnosis and risk assessment of hematological malignancies. By detecting genomic alterations often missed by conventional methods, OGM enhances diagnostic accuracy and prognostic evaluation. Its high resolution and comprehensive analysis make it a strong candidate for first-line cytogenetic assessment, potentially prompting updates in current risk stratification systems to reflect more detailed genomic information. When combined with NGS, OGM provides complementary genomic insights, thereby further enhancing the overall characterization and management of these diseases.

## 4. Materials and Methods

### 4.1. Patients

A total of 114 patients diagnosed at the Hospital Universitario Virgen de las Nieves, Granada, Spain, were included in the study. These patients were diagnosed with various oncohematological diseases according to the WHO and ICC classifications (Table 2) [1,2]. The distribution of pathologies included 43 patients diagnosed with AML, 4 with acute promyelocytic leukemia (APL), 54 with MDS, 11 with B-ALL, and 2 with T-cell acute lymphoblastic leukemia (T-ALL). The patients’ demographic data can be found in Table 2. All patients enrolled in this study were of Caucasian ethnicity.

All patient samples were collected according to local medical ethical regulations according to the Declaration of Helsinki.

### 4.2. Cytogenetics

Fluorescence in situ hybridization (FISH) techniques were used to evaluate the principal chromosomal alterations in MDS (del(5q), del(7q), del(17p)), AML (rearrangements of the *KMT2A* gene (11q23.3) and *CBFB* gene (16q21), and translocations such as *PML::RARA*, t(15;17)(q24;q21) and *RUNX1::RUNX1T1*, t(8;21)(q22;q22)), and ALL (rearrangement of the *KMT2A* gene (11q23.3) and *BCR::ABL1*, t(9;22)(q34;q11); *ETV6::RUNX1*, t(12;21)(p13;q22), and/or *PBX1::TCF3*, t(1;19)(q23;q22) translocations). Analyses were performed using conventional interphase techniques with the use of commercially available specific probes (MetaSystems Probes GmbH, Altlußheim, Baden-Württemberg, Germany).

Karyotype analysis of patients diagnosed with AML, MDS, and ALL was performed at an external laboratory.

### 4.3. Optical Genome Mapping

High-molecular-weight DNA (>250 Kb) was extracted using the Bionano Prep SP-G2 BMA/PB DNA Isolation Kit (Bionano Genomics Inc., San Diego, CA, USA), following the instructions provided in the protocol. Long-chain DNA was labeled with a fluorophore through the action of the DLE-1 enzyme, which recognizes the specific 6-nucleotide sequence (CTTAAG) repeated throughout the genome (Bionano Prep Direct Label and Stain (DLS) Kit; Bionano Genomics Inc., San Diego, CA, USA). The labeled DNA molecules were loaded onto a chip, linearized, and scanned using the Saphyr system (Bionano Genomics Inc., San Diego, CA, USA). The scanning process generated a digitalized image with a labeling pattern that was compared against the human reference map, enabling the visualization and characterization of structural variations and copy number variations present in the DNA under study.

Analysis of the results was conducted using Bionano Access software (Rare Variant Analysis algorithm), version 1.8.1 (Bionano Genomics Inc., San Diego, CA, USA), with the reference genome version GRCh38. During the analysis, confidence filters recommended by the manufacturer were applied, and benign or polymorphic structural variants, as well as structural alterations smaller than 200 Kb, were filtered out, except for those affecting genes relevant to the pathology. Various databases were utilized in the analysis of results, including the Atlas of Genetics and Cytogenetics in Oncology and Cancer and the Chromosome Aberrations and Gene Fusions in Cancer-The Mitelman Database (National Cancer Institute, Bethesda, MD, USA).

## Figures and Tables

**Table 1:** Clinical Data and summary of results from karyotyping, FISH, and OGM in a subset of studied patients.

Patient ID	Sex	Age	WHO 2022 Diagnosis	Gene Variant by NGS (VAF%)	Karyotype	FISH	SVs/CNVs Detected by OGM (ISCN 2024)
**1**	M	83	AML myelodysplasia-related	*BCOR* p.E1030Kfs*25 (82.9%) *U2AF1* p.S34F (42.3%) *DNMT3A* p.R882H (45.5%) *TET2* p.E1879Gfs*13 (43.1%) *TET2* p.M695Vfs*16 (43.6%) *KRAS* p.G12A (10.3%)	Normal	Normal	Clinically relevant/recurrent alteration: ***KMT2A*-PTD** (ogm[GRCh38] dup(11)(q23.3q23.3)(118,450,866_118,479,068)[0.86])
**2**	M	81	MDS with low blasts	*SF3B1* p.K700E (28.7%) *EZH2* c.1673-1G>A (26.5%) *ZBTB7A* )x3p.N532Kfs*8 (19.8%)	46.XY.add(20)(p1?)[21]/46.XY[15]	Normal	Clinically relevant/recurrent alteration: **7q22 deletion** (ogm[GRCh38] 7q22.1(100,242,336_102,318,833)x1[0.28],9p24.3p12(14,566_39,703,663 [0.53],20p13p11.1(70,156_26,271,555)x1[0.47])
**3**	M	25	AML with *CBFB*::*MYH11* fusion	*NRAS* p.Q61R (45%)	46,XY,del(16)(q22)[19]/46,XY[1]	del16q22.1 (*CBFB*)	Clinically relevant/recurrent alteration: ***MYH11*::*CBFB* fusión** ogm[GRCh38](16:16)(p13.11;q22.1)(15,709,259;67,079,181) (*MYH11*::*CBFB*)[VAF0.44],16q22.1(67,071,398_68,041,480)x1[0.68]
**4**	F	5	AML with *NUP98* rearrangement	*WT1* p.S381Lfs*71 (42.8%) *FLT3* p.G613_L656ins14 (8.6%) *FLT3* p.Y597_E598ins98 (4.8%) *FLT3* p.D835H (11.6%) *KRAS* p.G12V (9.9%)	Normal	Normal	Clinically relevant/recurrent alteration: NUP98::NSD1 fusion (ogm[GRCh38]t(5;11)(q35.3;p15.4)(177,215,724;3,743,680)(NUP98::NSD1)[VAF0.34])
**5**	F	80	MDS with low blasts	*TP53* p.Y234C (3.4%)	Normal	5q31 deletion (*EGR1*) monosomy 7	**Chromoanagenesis** involving interchromosomal translocations among chromosomes 4, 7, 12, and 21, accompanied by **monosomy 7, deletion of 5q, and *TP53* deletion.** (ogm[GRCh38] 4p16.3q27(1,848,033_121,936,972)x1[0.26], t(4;7)(q12;q21.11)(52,586,829;82,653,107)[0.14],t(4;21)(q12;q11.2) (58,287,437;14,614,511)[0.11],t(4;7)(q27;p14.1)(121,923,635;37,112,066)[0.12], 5q21.3q34(106,610,421_161,853,587)x1[0.24],(7)x1[0.25],t(7;12)(p14.2;q24.33) (36,347,679;130,801,616)[0.15],t(7;10)(q22.1;q24.1)(102,171,679;97,476,480)[0.1], t(7;21)(q34;q21.1)(141,134,229;15,771,635)[0.15],t(12;21)(q23.3;q21.1) (104,794,507;15,528,087)[0.16],12q23.3q24.33(104,987,455_130,581,372)x1[0.24], 17p13.1(7,642,059_8,781,416)x1[0.19])
**6**	M	12	Early T-ALL	*KRAS* p.G12V (39.4%) *NOTCH1* p.Q1584Pfs*33 (14.4%) *NOTCH1* p.R1586Afs*24 (14.5%) *NOTCH1* p.F1592S (10.6%)	Normal	Normal	Clinically relevant/recurrent alteration: ***CDKN2A/B* deletion, *TAL1*::*TRD* fusion** (ogm[GRCh37] t(1;14)(p33;q11.2)(47,693,608;22,897,900)[0.58], 9p21.3p13.1(21,763,632_38,783,625)x1[0.9],t(9;16)(p21.3;q23.1) (21,763,632;75,644,791)[0.73],9p24.3p23(14,566_ 12,148,131)x1[0.9], 9q21.11q34.3(70,321,158_138,832,483)x3[0.8],16q21q24.2 (65,347,021_88,205,172)x3[0.9],t(16;17)(q21;q25.3)(65,359,297;81,194,161)[0.58])
**7**	M	6	B-ALL with high hyperdiploidy	*PTPN11* p.A72T (4.6%)	No dividing cells	Normal	Clinically relevant/recurrent alterations: ***IKZF1* deletion, hyperdiploidy** (ogm[GRCh38] (X)x2[0.7],1q21.1q44(144,201,874_ 248,943,333)x3[0.7], (4-6)x3[0.7],7p22.3p11.2(2,496,413_58,027,799)x1[0.74], 7q11.1q36.3(61,875,656_ 158,250,255)x3[0.78],(8)x3[0.7], 9p24.3p12(14,566_39,918,350)x3)[0.8],(10)x4[0.4],(11,14,17)x[0.7],(18,21)x4[0.4])
**8**	M	1	B-ALL with other defined genetic abnormalities	*SH2B3* p.R195Qfs*57 (37.9%) *PAX5* p.T75R (35.1%)	46,XY,del(9)(p21),+10,−20[4]/46,XY[36]	Normal	Clinically relevant/recurrent alteration: ***CDKN2A/B* deletion** (ogm[GRCh38]9p24.3p13.1(14566_38,885,219)x1[0.88],(10)x3[0.4], 20q11.21q13.33(32,666,049_61,861,320)x1[0.9])
**9**	M	9	ALL with iAMP21	*SH2B3* p.D231Gfs*39 (92.9%) *PTPN11* p.A27T (45.3%)	Normal	**21q22 amplification** (***RUNX1***)	Clinically relevant/recurrent alterations: **iAMP21, deletions of *CDKN2A/2B, ETV6,* and *ERG.*** ogm[GRCh38] 8p23.3p12(61,805_33,511,396)x1, 9p24.3p13.2(14,566_36,586,614)x1[0.1],9q21.11q34.2(67,387,240_133,526,602)x3[0.4], 12p13.2p13.1(11,654,206_14,580,564)x1[0.9],21q21.1q22.3(20,026,617_42,737,810) amp[0.5],21q22.2(38,410,326_38,585,696)x1[0.15])
**10**	F	6	Li-Fraumeni syndrome/ B-ALL with high hyperdiploidy	*FLT3* p.D835H (13.1%) *KRAS* p.G13D (14.2%) *KRAS* p.G12D (9.3%) *KRAS* p.Q61H (5.1%) Germline *TP53* p.T125M (48%)	56.XX.+X.+4.+6.+8.+10.+12.+17.+18.+19. +21[4]/46.XX[46]	Normal	Clinically relevant/recurrent alterations: **Hyperdiploidy, *CDKN2A/2B* deletion** ogm[GRCh38] (X)x3[0.9],(4)x3[0.8],(6,8)x3[0.9], 9p21.3(21,960,511_22,019,278)x1[0.82],(10,14,17,18)x3[0.9],(21)x4[0.9]

WHO: World Health Organization [1,2]; NGS: next-generation sequencing; VAF: variant allele frequency; FISH: fluorescence in situ hybridization; OGM: optical genome mapping; SV: structural variant; CNV: copy number variant; ISCN: International System for Human Cytogenetic Nomenclature 2024; M: male; F: female; AML: acute myeloid leukemia, MDS: myelodysplastic syndrome, B-ALL (B-acute lymphoblastic leukemia; T-ALL (T-acute lymphoblastic leukemia; N/A: not available; ND: not detected. * In FISH study, probes were used to analyze recurrent and/or clinically relevant alterations in AML/MDS (rearrangements of KMT2A (11q23.3) and CBFB (16q21); PML::RARA, t(15;17)(q24;q21) and RUNX1::RUNX1T1, t(8;21)(q22;q22) translocations; TP53 deletion (17p13.3); 5q deletion (EGR1 [5q31]) and 7q deletion (KMT2E [7q22.3], CUL1/EZH2 [7q36.1], and the D7Z1 locus)); and in ALL (KMT2A rearrangements (11q23.3) and the BCR::ABL1, t(9;22)(q34;q11), ETV6::RUNX1, t(12;21)(p13;q22), and/or PBX1::TCF3, t(1;19)(q23;q22) translocations. According to ISCN 2024, formulas considered CNVs >5 Mb and SVs >200 kb, provided they did not affect clinically relevant regions, as well as interchromosomal and intrachromosomal translocations [14]. The FISH images of patients 2 and 9 are included in Supplementary Figure S1.

**Table 2:** Patient demographics by diagnosis: sex distribution and age range.

Diagnosis	Women (%)	Men (%)	Age; Median Years (Range)
SMD	20 (37)	34 (63)	65.1 (15–90)
AML	21 (49)	22 (51)	62.9(13–91)
Pediatric B-cell ALL	2 (33.3)	4 (66.6)	5.2 (1–9)
Adult B-cell ALL	2 (40)	3 (60)	35.6 (15–68)
T-cell ALL	0	2 (100)	18 (12–24)
APL	2 (50)	2 (50)	45.5 (13–67)

ALL: acute lymphoblastic leukemia; AML: acute myeloid leukemia; APL: acute promyelocytic leukemia; SMD: myelodysplastic syndrome.

## Data Availability

The data supporting the findings of this study are available from the corresponding author upon reasonable request.

## Supplementary Materials

The following supporting information can be downloaded at: https://www.mdpi.com/article/10.3390/ijms26125763/s1.
