# Supplementary material for: Application of Optical Genome Mapping for the Diagnosis and Risk Stratification of Myeloid and Lymphoid Malignancies
Source: Int J Mol Sci. 2025 Jun 16;26(12):5763. doi: 10.3390/ijms26125763 (PMC12192876; doi:10.3390/ijms26125763)
Supplement: Supplementary file 1 [file ijms-26-05763-s001.zip › Supplementary Figure 1.pptx]

## Slide 1
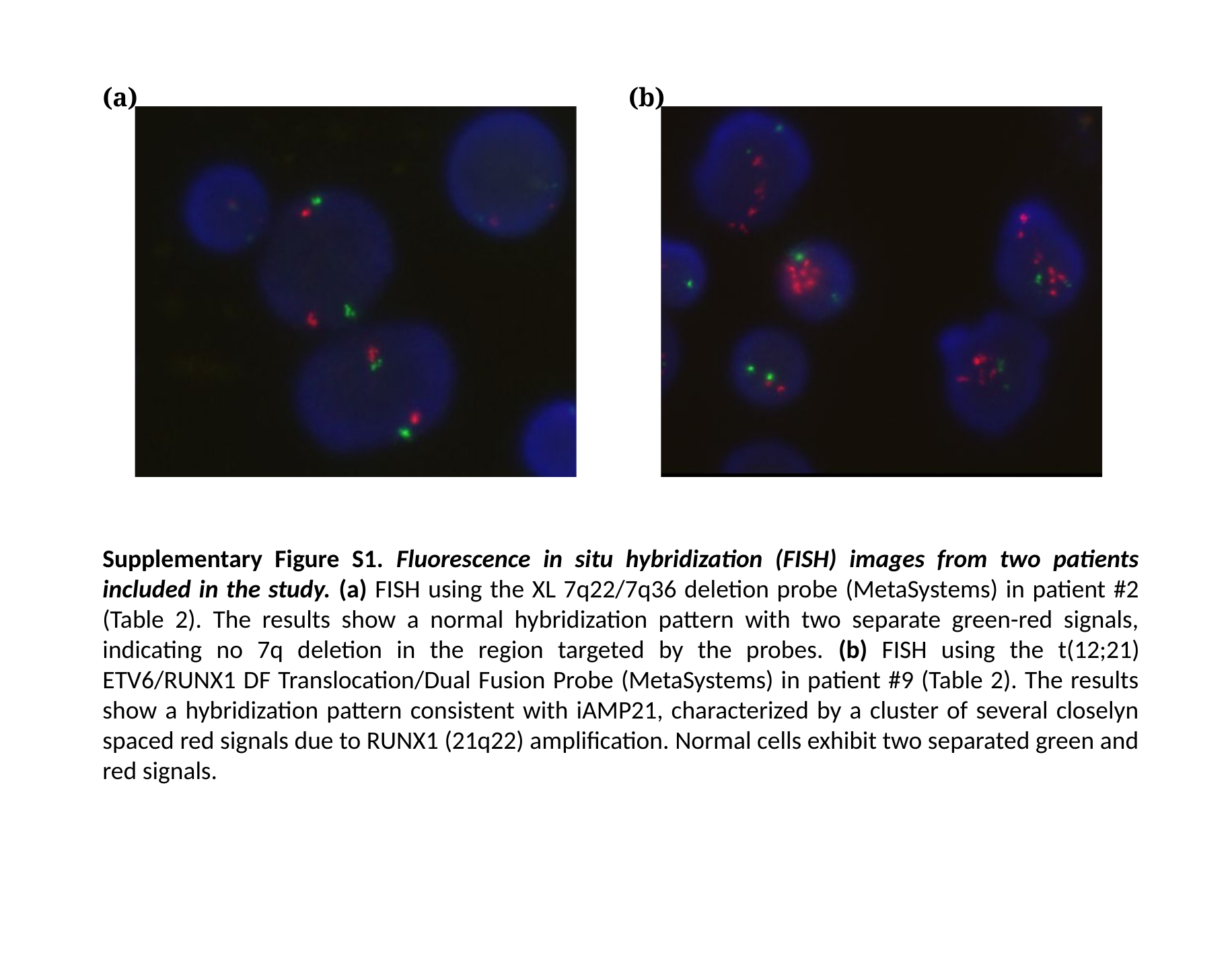

(a)
(b)
Supplementary Figure S1. Fluorescence in situ hybridization (FISH) images from two patients included in the study. (a) FISH using the XL 7q22/7q36 deletion probe (MetaSystems) in patient #2 (Table 2). The results show a normal hybridization pattern with two separate green-red signals, indicating no 7q deletion in the region targeted by the probes. (b) FISH using the t(12;21) ETV6/RUNX1 DF Translocation/Dual Fusion Probe (MetaSystems) in patient #9 (Table 2). The results show a hybridization pattern consistent with iAMP21, characterized by a cluster of several closelyn spaced red signals due to RUNX1 (21q22) amplification. Normal cells exhibit two separated green and red signals.
