# Supplementary material for: Application of Optical Genome Mapping for the Diagnosis and Risk Stratification of Myeloid and Lymphoid Malignancies
Source: Int J Mol Sci. 2025 Jun 16;26(12):5763. doi: 10.3390/ijms26125763 (PMC12192876; doi:10.3390/ijms26125763)
Supplement: Supplementary file 1 [file ijms-26-05763-s001.zip › Supplementary Table 1.pdf]

Supplementary Table S1. Analysis of Copy Number Variants (CNVs) using Next-Generation Sequencing (NGS)

ID Patient	Gene	Chromosomal Band	CN Status*	Compatible with
1	*KMT2A*	11q23	Gain (2.8)	11q23 gain
2	*CUX1*	7q22	Deletion (1.4)	7q22 deletion
	PAX5, CDKN2A, JAK	9p24p13	Gain (2.6)	9p24p13 gain
3	*CTCF*	16q22	Deletion (1.1)	16q22 deletion
4	Normal Copy Number Variation Analysis			
5	Normal Copy Number Variation Analysis			
6	*NRAS*	1p13	Deletion (1.1)	1p13 deletion
	PAX5, CDKN2A, JAK2	9p24p13	Deletion (1.1)	9p13–9p24 deletion
	HNRNPK NOTCH1, ABL1	9q21q34	Gain (2.7)	9q21–9q34 gain
	RBBP6	16p12	Deletion (1.8)	16p12 deletion
	CTCF, FANCA	16q22q24	Gain (3.0)	16q22q24 gain
7	*DHX15*	4p15	Gain (2.8)	Trisomy 4
	PDGFRA, KIT, SFRP2, TET2	4q12q24	Gain (2.8)	
	TERT	5p15	Gain (2.8)	Trisomy 5
	CSNK1A1, DDX41, NPM1	5q32q35	Gain (2.8)	
	IKZF1	7p12	Deletion (1.2)	IKZF1 deletion
	SBDS, SAM9D, CUX1, MET. BRAF, LUC7L2, EZH2	7q21q36	Gain (2.8)	7q gain
	MYC, RAD21, SMAD1	8q22q24	Gain (2.8)	Trisomy 8
	PAX5, CDKN2A, JAK2	9p24p13	Gain (2.8)	9p gain
	ANKRD26, SMC3	10p25p10	Gain (3.6)	Tetrasomy 10
	WT1, HRAS	11p15p13	Gain (2.8)	Trisomy 11
	ATM, KMT2A, CBL	11q22q23	Gain (2.8)	
	TP53	17p13	Gain (2.9)	Trisomy 17
	NF1, STAT3, STAT5B, PPM1D, SRSF2	17q11q25	Gain (2.9)	
	SETBP1	18q12	Gain (3.6)	18q12 gain
	RUNX1, U2AF1	21q22	Gain (3.6)	21q22 gain
	GATA1, KDM6A, SMC1A, PIGA, ZRSR2	Xp22p11	Gain (1.8)	X chromosome gain

	ATRX, STAG2, PHF6, BCORL1, BRCC3	Xq21q28	Gain (1.8)	
8	*PAX, CDKN2A, JAK2*	9p24p13	Deletion (1.1)	9p deletion with biallelic loss of *CDKN2A*
	ANKRD26	10p12	Gain (2.3)	Trisomy 10
	SMC3	10q25	Gain (2.3)	
	GNAS, RTEL1	20q13	Deletion (1.2)	20q deletion
9	*CSMD1*	8p23	Deletion (1.0)	8p23 deletion
	CDKN2A	9p21	Deletion (0.0)	9p deletion with biallelic deletion of CDKN2A
	HNRNPK NOTCH1, ABL1	9q21q34	Gain (2.9)	9q21q34 gain
	ETV6	12p13	Deletion (1.0)	12p13 deletion with heterozygous deletion of *ETV6*
10	*DHX15*	4p15	Gain (2.9)	Trisomy 4
	PDGFRA, KIT, SFRP2, TET2	4q12q24	Gain (2.9)	
	CSMD1	8p23	Gain (3.0)	Trisomy 8
	MYC, RAD21	8q24	Gain (3.0)	
	CDKN2A	9p21	Deletion (1.0)	9p21 deletion
	ANKRD26	10p12	Gain (2.6)	Trisomy 10
	SMC3	10q25	Gain (2.6)	
	TP53	17p13	Gain (2.8)	Trisomy 17
	NF1, STAT3, STAT5B, PPM1D, SRSF2	17q13q25	Gain (2.8)	
	SETBP1	18q12	Gain (2.9)	18q12 gain
	RUNX1, U2AF1	21q22	Gain (3.9)	21q22 gain
	GATA1, KDM6A, SMC1A, PIGA, ZRSR2	Xp22p11	Gain (2.9)	Trisomy X
	ATRX, STAG2, PHF6, BCORL1, BRCC3	Xq21q28	Gain (2.9)	

*The number in parentheses represents the copy number fraction detected in the specified regions.

Gene fusion detection using targeted Next-Generation Sequencing (NGS) panels

ID Patient	5' Fusion Gene	5' Transcript RefSeq ID	3' Fusion Gene	3' Transcript RefSeq ID	% Reads
4	*NUP98*	NM_016320:12	*NSD1*	NM_022455:6	28.73

% Reads: Percentage of mapped reads in which the rearrangement has been detected, relative to the total mapped reads in the sample
